# Generics and Alternatives

**DOI:** 10.3389/fpsyg.2020.01274

**Published:** 2020-07-03

**Authors:** Arnold Kochari, Robert Van Rooij, Katrin Schulz

**Affiliations:** Institute for Logic, Language and Computation, University of Amsterdam, Amsterdam, Netherlands

**Keywords:** generics, alternatives, probabiltiy, semantics, experiments

## Abstract

In this paper we argue that for the (probabilistic) interpretation of generic sentences of the form “*G*s are *f*,” three types of alternatives play a role: (i) alternative features of *f*, (ii) alternative groups, or kinds, of *G*, and (iii) alternative causal background factors. In the first part of this paper we argue for the relevance of these alternatives. In the second part, we describe the results of some experiments that empirically tested in particular the second use of alternatives.

## 1. Introduction

Bare plural (or BP) generic sentences like “Birds fly” and “Tigers are striped” (which we take to have the form “*G*s are *f*”) are sentences that, by their very nature, express useful generalizations. Accounting for the meaning of these sentences has been proven to be notoriously difficult. The problem is to account for the fact that generics allow for exceptions. We believe that birds fly, even though not all birds do or can fly.

One very popular solution to this problem proposed in the linguistic literature is to assume the presence of a generic operator, which is then analyzed as a universal quantifier with a restricted domain of quantification: for the generic to be true all the *relevant* or *normal* members of the group *G*, or all the members under *normal* circumstances, have to have the feature *f* under discussion (e.g., all relevant or normal birds fly, or all objects being birds under normal circumstances fly) (cf. Asher and Morreau, 1995). But without an independent and satisfying account of what relevance and normalcy is this will not bring us any closer to a true solution of the problem.

We will follow here a different line of approach to the meaning of generic sentences. This is the idea that their meaning should be related to the frequency with which we observe a member of the group *G* to bear feature *f*. A very natural and often explored approach along these lines is the majority rule for the interpretation of generics (Cohen, 1999, 2004). According to the majority rule a generic is true in case the probability of a member of group *G* having feature *f* is high, (much) higher than $\frac{1}{2}$.

**Definition** 1. *A simple majority rule for generics*.

*A generic sentence* ‘*Gs are f' is true in case* $P\left(f|G\right)>\frac{1}{2}$.

Thus, taking a generic like (1), according to definition 1 this sentence is true in case the majority of the birds fly.

(1) Birds fly.

This natural approach to the meaning of BP generics nicely accounts for the fact that not all birds need to fly in order for the generic to be true and still plays an important role in the literature on generic expressions. But while it has been shown that frequency does play a role for the meaning of generics (e.g., Prasada and Dillingham, 2006), this approach has difficulties to account for the different degree with which generics allow for exceptions. In some cases we are willing to accept generic sentences even in cases where only very few group members carry the feature in question. For instance, a generic statement like (2) is generally accepted to be true, even though only 1% of mosquitoes are actually carriers of the virus (Cox, 2004).

(2) Mosquitoes carry the West Nile virus.

There are many more studies that enforce the conclusion that the truth of a generic sentence cannot be in general reduced to a high conditional probability of *f* on *G*. Experimental evidence was first provided by Gilson Gilson and Abelson (1965), but similar conclusions also emerged in the linguistic literature (e.g., Lawler, 1973; Dahl, 1975; Carlson, 1977; Declerck, 1986). These results were then confirmed in psychological studies (e.g., Prasada, 2000; Gelman, 2004; Gelman and Bloom, 2007; Cimpian et al., 2010a).

Especially in the psychological literature on generics the observation that the meaning of generics cannot be reduced to a high conditional probability has then been taken to show that there is no systematic relation between the meaning of generics and statistical information (e.g., Leslie, 2008; Cimpian et al., 2010b). This conclusion is wrong in our eyes, or at least premature. The fact that Rule 1 is not an adequate description of the truth conditions of generic sentences does not show that no statistically based rule for the meaning of generics is possible—as claimed by the authors mentioned above. More concretely, in this paper we will show that if we take into account alternatives we can substantially improve on Rule 1 and can account for examples like (2).

We will argue for an extension of this rule involving three sets of alternatives.

1. Alternatives of the property *f*, *Alt*(*f*), limit the domain of the probability function involved in the evaluation of the generic statement.
2. Alternatives of the group *G* the generic statement is about, *Alt*(*G*), help to determine to what extent *f* is a *distinctive* feature for group *G*.
3. Alternatives, in the sense of causally background factors, influence our assessment of the extent to which (being a) *G* is causally relevant to *f*.

By taking the second and third type of alternatives very seriously, we will end up with an interpretation rule of which the majority rule is only a special case. The third set of alternatives also provides a straightforward link to experimental results showing that there is a close relation between judgements concerning generic sentences and general causal knowledge about the world (Murphy and Medin, 1985; Murphy, 2004). Again, we will argue here that a causal approach to generics should not be seen as a competitor to the statistical approach, but that both approaches are closely related (in contrast, for instance, to what is claimed in Cimpian et al., 2010b).

Our argumentation will proceed step-wise, starting with the first set of alternatives in section 2.1, continuing with the second set in section 2.2, and finally introducing the third notion of alternatives in section 2.3. So, in the first part of the paper each section will end with a new, extended version of the majority rule just introduced. In the second part of the paper we will zoom in on the second type of alternatives we have added and provide additional support for our claim that they play a role for the interpretation of generics. In section 3.1, we will connect our approach to BP generics to the analysis of conditioning in the psychology of learning. This leads to a last adaption of the approach to generics defended here, introduced in section 3.2. In section 4, we will present the results of two experiments testing this approach to BP generics.

## 2. The Different Ways Generics Depend on Alternatives

### 2.1. Alternatives to Determine the Probability Domain

The most straightforward way to link the truth conditions of generic sentences to statistical data is the majority rule introduced in section 1: to account for the truth of (1) we demand that the *majority* of birds fly. We already discussed in the introduction an example showing that such an account doesn't work in general. Examples like (3-a) and (3-b) make the same point. Again, these generics are acceptable, even though *P*(*f*|*G*) seems to be less than half.

(3)  a.  Ducks lay eggs.

       b.  Goats produce milk.

However, these examples can be given a majority analysis after all, if we make an additional use of alternatives (cf. Cohen, 1999). The relevant alternatives for a generic of the form “*G*s are *f*” will be alternatives to the feature *f*, i.e., *Alt*(*f*). For (3-a), for instance, we should take into account *Alt*(*lay eggs*). Intuitively, *Alt*(*lay eggs*) will consist of alternative ways of reproduction. Thus, *Alt*(*lay eggs*) = {*lay eggs, give live birth*}. Cohen (1999) proposes that the probability function relevant for the interpretation of the generics should now not range over *all* objects, but be restricted to the set of objects that satisfy at least one of the properties in *Alt*(*f*), i.e., ⋃*Alt*(*f*). We end up with the following adaption of our stable majority rule.

**Definition** 2. *Truth conditions for generics with *Alt*(*f*) alternatives. A generic sentence “*Gs are f”* is true in context *c* in case for the contextually salient set *Alt*(*f*) of alternatives to *f* it holds that*

$$P\left(f|G\cap ⋃Alt\left(f\right)\right)>\frac{1}{2}.$$

Because ⋃*Alt*(*lay eggs*) ≈ *Females*, a majority analysis could, or would, predict that (3-a) is true just in case a (stable) majority of *female* birds lay eggs^1^.

Unfortunately, as already known by Cohen (1999), definition 2 won't do. There are various other examples where this application of alternatives won't save the majority rule. In general, a high conditional probability of *f* given *G* appears to be neither a *sufficient*, nor a *necessary* condition for the corresponding generic to be true. As for necessity, it is unclear how even the new Definition 2 could explain example (2) from the introduction, or example (4), which is very similar to (3-a) and (3-b).

(4) Ducks have colorful feathers.

The following type of examples, mostly due to Carlson (1977), have been used to show that a high conditional probability is not a sufficient condition either:

(5)  a.  *Chicken are female.

    b.  ?Chinese speak Mandarin.

    c.  ?People are over 3 years old.

    d.  ?Crocodiles die before they attain the age of 2 weeks.

    e.  ?Primary school teachers are female.

    f.  ?Bees are sexually sterile.

Although these generic sentences all seem false, or at least not (obviously) true, their corresponding conditional probabilities are high. In particular, although about 80% of all chicken are female, due to the fact that, for economic reasons, most farmers gas male chicks immediately after birth, the generic (5-a) seems false. In all these cases the amended majority rule proposed in Definition 2 is of no help. A similar point can also be made with the following two famous examples.

(6)    a.    ?Books are paperbacks.

     b.    ?Mammals are placental mammals.

Again,the approach fails, because most naturally,

⋃Alt(paperbacks)=⋃{paperbacks,hard-covers}⊆Books,

with the result that (6-a) is still falsely predicted to be true, if the majority of books are paperbacks. But there might be another way to go. Perhaps we can demand that for a generic of the form “*G*s are *f*” to be appropriate, it cannot be the case that ⋃*Alt*(*f*) ⊆ *G*. This constraint would immediately rule out examples like (6-a) and (6-b) and some other weird generics like “Humans are autistic,” which would be predicted to be inappropriate, instead of just false, simply because only humans can be autistic (or let us assume so). This constraint certainly helps with some of the counterexamples to sufficiency. But it is of little help when it comes to examples like (5-a). Additionally, so far we miss a rationale behind this constraint, though we will provide one in section 3.2.

In the following section, we will discuss the use of two more sets of alternatives in the definition of the truth conditions of generic sentences. The first set will be used to account for the examples that show that high conditional probability is not a necessary condition for the truth of a generic. The second set will be used to explain why it is not a sufficient condition either.

### 2.2. Subject Term-Alternatives and Relative Readings

Let's have a look at a different class of very famous examples. Much ink has been spilled on the following “Port-Royal” type of generics:

(7)    a.   Dutchmen are good sailors;

    b.   Bulgarians are good weightlifters.

Intuitively, the above sentences are appropriate, although only a small percentage of Dutchmen are good sailors and only few of all Bulgarians are good weightlifters. It is also not the case that limiting the domain of the probability function to ⋃*Alt*(*good sailor*) would make (7-a) true on a majority analysis after all, because naturally ⋃*Alt*(*good sailor*) could include also things like “soldiers,” “(good) peasants,” etc. One can imagine several strategies to deal with such sentences^2^. For instance, one might propose that limiting the domain to ⋃*Alt*(*good sailor*) would still do: Because in a natural use of (7-a) the adjective ‘good' typically is stressed, the set *Alt*(*good sailor*) would typically be just {*good sailors, moderate sailors, bad sailors*}. Thus, ⋃*Alt*(*good sailors*) = *Sailors*, meaning that the domain of the probability function would range only over sailors. It follows that (7-a) is predicted to be true on a majority analysis just in case most Dutch sailors are good sailors.

This solution, however, appears to be not particularly convincing. The reason is that although Bulgarian weightlifters are pretty successful at the olympics, it is questionable whether most Bulgarian weightlifters are good weightlifters. Similarly, it is questionable whether most Dutch sailors are (or were in the seventeenth century) good sailors. A much more natural solution seems to be to propose (perhaps with Nickel, 2012) that (7-a) is true just because the good Dutch sailors are good compared to good sailors in general *and* the moderate Dutch sailors are good compared to moderate sailors in general *and* the bad Dutch sailors are good compared to bad sailors in general. Interestingly, this reading is close to Cohen's (1999) analysis of sentences like (7-a) as *relative readings* of generics.

Cohen (1999) proposed that generics like (7-a)–(7-b) are true, because they should be interpreted differently than standard generics, namely in a *relative* way: (7-a) is true iff compared to relevant alternative people in the seventeenth century (Frenchmen, Spaniards, Englishmen, and people from the Germanic countries), *relatively many* Dutchmen are good sailors. Similarly for (7-b). In probabilistic terms this means that *P*(*f*|*G*) > *P*(*f*)—or better *P*(*f*|*G*∩ ⋃*Alt*(*f*)) > *P*(*f*|⋃*Alt*(*f*))—should hold with “*G*” denoting the Dutchmen and “*f*” standing for “are good sailors.' “Making use of relative readings, we could also account for the fact that examples like (4) are, intuitively, true.

Cohen (1999) links the two readings of generic sentences to particular intonation patterns of the sentence used. If in the use of a generic sentence of the form “*G*s are *f*” it is the feature *f* that is stressed by intonation, the generic sentence will have a standard (stable) majority reading. But if (topical) stress is given to the subject term “*G*,” the relative reading follows. It is standardly assumed that topical stress indicates a contrast between that what is stressed, and the alternatives of the stressed item. The stress on *G* then indicates a contrast with denotations of other terms *G*_1_, ⋯ , *G*_*n*_: compared to the alternatives of *G*, i.e., *G*_1_, ⋯ , *G*_*n*_, many *G*s have feature *f*. This suggests that the generic “*G*s are *f*” is true in that case only if ∀*i*:*P*(*f*|*G*) > *P*(*f*|*G*_*i*_), or perhaps, only if *P*(*f*|*G*) > *P*(*f*|⋃{*G*_1_, ⋯ , *G*_*n*_})^3^. If we assume that the “domain” of the probability function is *G*∪⋃{*G*_1_, ⋯*G*_*n*_} and that *G* is incompatible with all the *G*_*i*_, the latter suggestion comes down to the requirement for “*G*s are *f*” to be true that *P*(*f*|*G*) > *P*(*f*|¬*G*). Interestingly enough, it can be easily proved that *P*(*f*|*G*) > *P*(*f*) if and only if *P*(*f*|*G*) > *P*(*f*|¬*G*), and thus that “*G*s are *f*” is true on Cohen's relative reading exactly if *P*(*f*|*G*) > *P*(*f*|¬*G*). Hence, we can derive the relative meaning from a more general and independently motivated approach to the interpretation of focus.

Taking all that has been said about the relevance of alternatives for the meaning of generics into account, we end up with the following definitions of the truth conditions of generic sentences.

**Definition** 3. Truth conditions for generics with *Alt*(*f*) and *G*-alternatives.

A generic sentence “*Gs are f”* is ambiguous between an **absolute** and a **relative reading**. In its absolute reading the conditions of Definition 2 apply. In its relative reading the generic is true, in context *c* in case for a contextually salient set *Alt*(*f*) of alternatives to *f* and a contextually salient set *Alt*(*G*) of alternatives to *G* it holds that

 P(f|G∩⋃Alt(f))>P(f|⋃Alt(G)∩⋃Alt(f)).

Suppose that a generic has a relative reading. In that case it is clear that high conditional probability is not a sufficient condition for the corresponding generic to be true. For instance, it might be that although *P*(*f*|*G*) is high, still *P*(*f*|*G*) < *P*(*f*|¬*G*). Perhaps we could account for the falsity of the following sentences, by assuming that they receive a relative reading.

(8)     a.     *Chicken are female.

     b.  ?Chinese speak Mandarin.

     c.    ?People are over 3 years old.

     d.    ?Crocodiles die before they attain the age of 2 weeks.

     e.    ?Primary school teachers are female.

     f.    ?Bees are sexually sterile.

Although we think that it is quite natural that these sentences receive a relative reading, that won't help to predict all these sentences to be false: although it might explain why (8-c) is bad^4^, (8-b), for instance, would obviously be true on its relative reading as well.

To account for these type of examples, Cohen (1999) and Cohen (2004) proposes a *homogeneity condition*. Rather than just demanding (for the absolute reading) that *P*(*f*|*G*) is high^5^. Cohen demands that the conditional probability of *f* given a set of *G*s should be high for each cell of a contextually determined salient partition {*G*_1_, …, *G*_*n*_} of *G*. Thus, each of *P*(*f*|*G*_1_)⋯*P*(*f*|*G*_*n*_) should be high. Although it is not usually thought of in that way, each cell *G*_*i*_ could, in fact, be thought of as an alternative. Concentrating on (8-f), for instance, a salient partition of bees into queens (female), workers (female) and drones (male) will correctly predict that (8-f) is false, because neither queens nor drones tend to be sterile. Cohen provides a similar explanation for other examples as well.

We think this proposal is promising, and we are sympathetic to this proposal because making use of the homogeneity condition fits well with our idea that generic sentences express inductive generalizations about unbounded sets (cf. section 4). Still, Leslie (2008) has persuasively argued that the condition of homogeneity not only explains away bad generics, but good ones as well. Why, for instance, is “Bees reproduce” true on Cohen's salient partition of bees?^6^ More dramatically, consider (1) “Birds fly.” This generic is predicted to be false on both readings, if the relevant partition is a bi-partitioning of birds into Penguins, on the one hand, and all the other types of birds, on the other. Why is this partition not the relevant one? Of course, Cohen could claim that this partition is not the salient one with respect to which the sentences should be interpreted, but then the question is, why not?

### 2.3. Alternative Causal Background Conditions

In van Rooij and Schulz (2019, 2020b), we have argued that many generics should be given a *causal* analysis. It is not the conditional probability that should be high in order for a generic of the form “*G*s are *f*” to be true, it should rather be the case that having property *G* has a *significant causal impact* on also having feature *f*^7^. Intuitively, “*G*s are *f*” is true on this analysis, if being a *G*, or having property *G*, is causally sufficient (with high probability) for also having feature *f*. The notion of “*causal impact*” is defined by Pearl (2000) in terms of intervention, making use of causal models. Fortunately, we can reformulate (or test) this notion without making use of interventions by making use of alternatives.

In causal models there exists a difference between the probability of *C* conditional on the observation of *A* and the probability of *C* conditional on making *A* true by intervention. The former is modeled by standard conditionalization, *P*(*C*|*A*). The latter, however, is modeled by *P*(*C*|*do*(*A*)). Whereas, *P*(*C*|*A*) has a purely evidential reading, *P*(*B*|*do*(*A*)) has a causal one. An appealing way to illustrate the difference between *P*(*C*|*A*) and *P*(*C*|*do*(*A*)) is by making use of partitions (Skyrms, 1980; Pearl, 2000). According to standard probability theory, $P\left(C|A\right)=\underset{i}{\sum }\left[P\left(C|B_{i}\wedge A\right)\times P\left(B_{i}|A\right)\right]$, with {*B*_*i*_} any partition of the state space. Instead, $P\left(C|do\left(A\right)\right)=\underset{i}{\sum }\left[P\left(C|B_{i}\wedge A\right)\times P\left(B_{i}\right)\right]$, where the *B*_*i*_ are the maximally specific causally relevant background factors^8^. Notice that although in general *P*(*C*|*A*) ≠ *P*(*C*|*do*(*A*)), they come to the same if *A* is probabilistically independent of the issue of which causal background factor in fact holds, i.e., if for all *B*_*i*_, *P*(*B*_*i*_|*A*) is the same as *P*(*B*_*i*_).

In section 2.2, we have seen that according to Cohen (1999) “*G*s are *f*” is true on its relative reading iff *P*(*f*|*G*) − *P*(*f*) > 0, which is equivalent with *P*(*f*|*G*) − *P*(*f*|¬*G*) > 0 (where ¬*G* stands for ⋃*Alt*(*G*)). If we would say that “*G*s are *f*” is true iff having property *G* has a *positive causal impact* on also having feature *f*, this comes down to demanding that *P*(*f*|*do*(*G*)) − *P*(*f*|*do*(¬*G*)) > 0. This already shows that the relative reading is closely related to the causal reading of generics. In fact, Cohen's relative reading can be seen as a special case of our causal reading. To see this, notice that in terms of causal background factors, the condition *P*(*f*|*do*(*G*)) − *P*(*f*|*do*(¬*G*)) > 0 reduces to $\left[\underset{i}{\sum }P\left(f|G\wedge B_{i}\right)\times P\left(B_{i}\right)\right]-\left[\underset{i}{\sum }P\left(f|\neg G\wedge B_{i}\right)\times P\left(B_{i}\right)\right]>0$. If the issue {*G*, ¬*G*} is independent of the issue which causal background factors in fact hold, this, in turn, comes down to $\left[\underset{i}{\sum }P\left(f|G\wedge B_{i}\right)\times P\left(G|B_{i}\right)\right]-\left[\underset{i}{\sum }P\left(f|\neg G\wedge B_{i}\right)\times P\left(\neg G|B_{i}\right)\right]>0$, which reduces to Cohen's relative reading: *P*(*f*|*G*) − *P*(*f*|¬*G*) > 0.

We have stated above that the causal impact of *G* should not just be positive, but should rather be *significant* in order for the generic to be true: the difference should be *significantly* above 0. Thus, we end up with the following causal analysis of generics: ^9^

**Definition** 4. The generic sentence “*Gs are f”* is true iff

           $\underset{i}{\sum }\left[P\left(f|G\wedge B_{i}\right)\times P\left(B_{i}\right)\right]\gg \underset{i}{\sum }\left[P\left(f|\neg G\wedge B_{i}\right)\times P\left(B_{i}\right)\right]$, where {*B*_*i*_} is a partition of maximally specific causally relevant background factors.

Notice that each causal background factor *B*_*i*_ of the partition {*B*_*i*_} can be thought of as an alternative, in a similar way as each cell *G*_*i*_ of the salient partition {*G*_1_, ⋯*G*_*n*_} used in Cohen's homogeneity condition can. We don't know whether the causal background partition can replace Cohen's homogeneity condition, but if so, it would explain why the partition {*Penguins, other birds*} is not a good partition with respect to which “Birds fly” must be interpreted, if we (with Skyrms, 1980) additionally demand that ∀*B*_*i*_:*P*(*f*|*G*∧*B*_*i*_) ≥ *P*(*f*|¬*G*∧*B*_*i*_). In any case, we think that a causal analysis, and thus our causal alternatives, can help to explain why some of (8-a)-(8-f) are false.

Take an example like (8-b). Obviously, a large population of Chinese speak Mandarin, so *P*(*M*|*C*) is high, and much higher then *P*(*M*|¬*C*). But on our causal analysis, we must compare *P*(*M*|*C*∧*B*_*i*_) with *P*(*M*|¬*C*∧*B*_*i*_) for the *B*_*i*_ that are causally relevant for whether or not somebody speaks Mandarin. Whether or not you live in China, or communicate a lot with people that live in China, seems a natural candidate. But when *B*_*i*_ stands for “living in China,” the difference between *P*(*M*|*C*∧*B*_*i*_) and *P*(*M*|¬*C*∧*B*_*i*_) doesn't seem to be that high. On the other hand, *P*(*B*_*i*_|*C*) is high (and *P*(¬*B*_*i*_|*C*) is low) and very different from *P*(*B*_*i*_). Thus, there is a difference between the evidential impact, *P*(*M*|*C*) − *P*(*M*|¬*C*), on the one hand, and the causal impact, *P*(*M*|*C*∧*B*_*i*_) − *P*(*M*|¬*C*∧*B*_*i*_), on the other: whereas the former difference is high, the latter difference is (presumably) low. But that is enough to explain why (8-b) is false, if we assume that the generic has a causal interpretation.

Other examples can be explained (away) in similar ways. Consider for instance (8-e), “Primary school teachers are female.” This sentence is predicted to be false on a causal interpretation, because there doesn't seem to be any *B*_*i*_ that is causally relevant for being female such that *P*(*F*|*PST*∧*B*_*i*_) − *P*(*F*|¬*PST*∧*B*_*i*_) is high, though being a primary school teacher is still evidentially relevant for the most natural partition {*B*_*i*_}, i.e., the genetic makeup.

Before we conclude this excursion into causality, note that the analysis of generics we propose here combines a causal analysis of generics with a probabilistic approach. We want to highlight this because, as mentioned in the introduction, the shortcomings of the majority rule are sometimes interpreted as showing that a statistical approach of generics is doomed to fail (cf. Leslie, 2008; Cimpian et al., 2010b). However, this is fallacious reasoning. There are many more options that one can take when exploring statistically approaches than just the majority rule. And the observed connections between the truth conditions of generics and assumed causal dependencies can also be captured nicely with a statistical approach. We will come back to this point in the next section, when we discuss the relation of generics to associative learning.

Furthermore, notice that the approach proposed here can, for instance, also account nicely for some of the experimental data on the dependence of generics on causal world knowledge. Cimpian et al. (2010b) reports that generics based on biological features are judged true more often than generics based on more accidental features (having a broken leg, or having infected ears). The generics based on biological features were also assumed to imply a significantly higher probability of the feature in the group than generics based on accidental features. Such generics would also have a hard time passing the truth conditions proposed in Definition 4.

## 3. Generics as Learning Generalizations

In this section we will focus on the second sense in which generics take alternatives into account: alternatives to the group *G* the generic claim is talking about. The alternative set *Alt*(*f*) will be put aside for the moment. In the first subsection below we will show that the semantics proposed by Cohen for the relative reading is strongly related to how in Psychology associative learning is described. This leads to an interesting new perspective on the meaning of generic sentences: we should understand their meaning in terms of the conditions under which we would learn the expressed generalization. This would give a natural explanation for why theories of learning appear so relevant for the meaning of generic sentences.

However, in two important ways this perspective does not mesh well with the approach we finished section 2 with. First of all, learning is something that grows gradually with the experience of the learner. There is no clear cut-off point in contrast to what Definition 3 assumes for both readings of generics. Second, the results from learning motivate the relative, not the absolute reading of generics that Cohen postulates. These two considerations will lead us to formulate an alternative approach to generics in section 3.2. This is the approach that will then be tested in the final section of the paper (section 4).

### 3.1. Subject Term-Alternatives and Learning

In this section we argue that there is an important justification for assuming that generic sentences (also) have a relative reading, and thus that the subject alternatives *G*_1_, ⋯ , *G*_*n*_ matter for the interpretation of a generic sentence. In section 2, we have stated that generic sentences express, by their very nature, useful generalizations. This suggests that there is a close relation between the truth conditions of generic sentences, on the one hand, and the way we *learn* generalizations, on the other. Much psychological research on learning was done before the cognitive revolution in psychology, in classical conditioning. In classical conditioning, what is learned is an association between a cue and an outcome. The cue, *c*, such as the sound of a bell, or a tuning fork, can become associated with an outcome, *o*, which can be thought of either as something like the taste of food, or a shock, or an unlearned reflex response to that, like salivation, or high blood pressure indicating fear.

What is the expectation that the *n* + 1th cue *c* will be accompanied with outcome *o*? The perhaps most natural idea would be that it is just the times that cue *c* was accompanied with outcome *o* divided by the times that cue *c* was given at all. If we say that *V*_*i*_(*o*|*c*) = 1 if at the *i*th exposure cue *c* is accompanied with outcome *o*, and that *V*_*i*_(*o*|*c*) = 0 if at the *i*th exposure cue *c* is not accompanied with outcome *o*, the expectation according to this natural idea that the *n* + 1th cue *c* will be accompanied with outcome *o*, i.e., $P_{n+1}^{*}\left(o|c\right)$, can be stated as follows:

$$\begin{array}{ll} \left(RF\right)\;P_{n+1}^{*}\left(o|c\right)\;=\frac{V_{1}\left(o|c\right)+\cdots +V_{n}\left(o|c\right)}{n}\\ = & \frac{1}{n}\overset{n}{\underset{i=1}{\sum }}V_{i}\left(o|c\right) \end{array}$$

It is well-known, however, that for the calculation of $P_{n+1}^{*}\left(o|c\right)$ it is not needed to maintain a record of all cases where cue *c* was accompanied with outcome *o*. One can calculate $P_{n+1}^{*}\left(o|c\right)$ incrementally as well, by constantly changing the expectations:

$$\begin{array}{lll} P_{n+1}^{*}\left(o|c\right)\; & =\; & \frac{1}{n}\overset{n}{\underset{i=1}{\sum }}V_{i}\left(o|c\right)\\ = & P_{n}^{*}\left(o|c\right)+\frac{1}{n}(V_{n}\left(o|c\right)-P_{n}^{*}\left(o|c\right)) \end{array}$$

It turns out that the form of this incremental learning rule is very common. It is known as learning by *expected error minimization* and is used in almost all modern methods of learning.

Although it is natural to think that the expectation of outcome *o* for the *n*+1th cue *c* will be $P_{n+1}^{*}\left(o|c\right)=\frac{1}{n}\overset{n}{\underset{i=1}{\sum }}V_{i}\left(o|c\right)$, this is not what is found experimentally, at least for animal learning. For animal learning, Rescorla (1968) observed that rats learn a tone (cue/cause)-shock (outcome) association if the frequency of shocks immediately after the tone is higher than the frequency of shocks undergone otherwise. This holds, even if in the minority of cases a shock actually follows the tone. Gluck and Bower (1988) and others show that humans learn associations between the representations of certain cues (properties or features) and outcome (typically another property or a category prediction) in a very similar way. Thus, we associate outcome *o* with cue *c*, not so much if *P*(*o*|*c*) is high, but rather if $\Delta P_{c}^{o}=P\left(o|c\right)-P\left(o|\neg c\right)$ is high, where $\Delta P_{c}^{o}$ is known as the *contingency* of *o* on *c*. How can this be explained? Rescorla and Wagner (1972) show that this can be explained by an error–based learning rule very similar to the one above. The only thing that really changes is that this time the learning rule is also *competition-based*. The idea is that a cue can also be taken as a *combination* of separate cues: if *c*_1_ and *c*_2_ are cues, *c*_1_*c*_2_ is taken to be a cue as well, and they all could be accompanied with the same outcomes. According to Rescorla and Wagner (1972), we should keep track of expectations, or associations, for cue-action pairs for all primitive cues, i.e., *c*_1_ and *c*_2_. For the calculation of $E_{n+1}^{*}\left(o|c_{1}\right)$ after the *n*th trial, however, we should also look at $E_{n+1}^{*}\left(o|c_{2}\right)$ in case the actual cue at the *n*th trial is the combined cue *c*_1_*c*_2_. The famous Rescorla-Wagner learning rule (RW) for each primitive cue *c*_*i*_ is stated as follows:

$$\left(RW\right)\;E_{n+1}^{*}\left(o|c_{i}\right)\;=\;E_{n}^{*}\left(o|c_{i}\right)+\lambda (V_{n}\left(o|c_{i}^{*}\right)-\underset{j}{\sum }E_{n}^{*}\left(o|c_{j}\right))$$

Here, $E_{n+1}^{*}\left(o|c_{i}\right)$ is the agent's expectation after *n* observations that the *n* + 1th primitive cue *c*_*i*_ has outcome *o*, where λ is a learning rate (typically very small) and where $V_{n}\left(o|c_{i}^{*}\right)$ measures the magnitude of the reinforcement at the *n*th trial where cue *c*_*i*_ was involved^10^. Although $E_{n+1}^{*}\left(o|c\right)$ converges to the actual conditional probability (or relative frequency) under some conditions, Cheng's (1997) shows that under most conditions $E_{n+1}^{*}\left(o|c\right)$ yields, instead, $\Delta P_{c}^{o}=P\left(o|c\right)-P\left(o|\neg c\right)$ in the long run (see also Danks, 2003). Thus, in those cases expectations, or associations, as generated by rule (RW) do not really measure probabilities, but contingency, instead^11^ We have noted already that $\Delta P_{c}^{o}=P\left(o|c\right)-P\left(o|\neg c\right)>0$ if and only if *P*(*o*|*c*) > *P*(*o*), i.e., the measure Cohen (1999) used to account for relative readings of generics. Interestingly, Yuille (2006) shows there exists a learning rule very similar to (RW) that converges to Cheng's (1997) notion of causal power, which is closely related with the notion of “causal impact” as discussed in section 2.3. Thus, not only Cohen's relative reading can be motivated through learning, the causal analysis of generics sketched in section 2.3 can be given a learning-theoretic motivation as well.

### 3.2. A New Proposal

Based on the discussion in the last section, we propose that the truth, or assertability, of generic sentences should be stated in terms of the conditions needed to learn the expressed generalization. More concretely, we want to propose (but see also van Rooij and Schulz, 2020a), that the measures used in the above discussed literature on learning can also be used to measure the assertability of generic sentences. To have a concrete measure to work with we take contingency, instead of the more general notion of causal impact. If for simplicity we also ignore the alternative set *Alt*(*f*), this gives the following proposal for the assertability of generic sentences.

**Definition** 5. *The assertability of a generic sentence “*Gs are f”* is given by the formula*

$$Assertability\;of{\;}^{\prime }Gs\;are\;f^{\prime }\;=\;P\left(f|G\right)-P\left(f|⋃Alt\left(G\right)\right).$$

We propose here that distinctiveness is at the heart of the meaning of generic sentences. Tessler and Goodman (2019) came up with a very similar proposal. Our motivation, however, is different: we propose Definition 5 because of the close connection between the meaning of generic sentences and how we learn (causal) generalizations. Definition 5 differs from the interpretation rule we ended up with in Definition 3 in that it replaces truth conditions for generics with degrees of assertability. We think that this is a step that we have to take. From a theoretical point the use of cut-off points seems necessary to allow for a truth-conditional approach to generics. This strategy to translate grades into a binary system occurs in semantics and philosophy of language at various points (vagueness, conditionals, etc.), but it is also known to be very problematic: a vague predicate is vague exactly because it does *not* seem to have a clear cutoff point. It doesn't seem to be convincing at all that we switch our ratings of assertability of sentences completely based on small differences in the frequencies that we observe. For similar reasons, and because of the link we want to make to associative learning, we propose here that at least the assertability of generics is a matter of degree. We don't want to engage in a discussion of what that would mean for truth conditionals semantics in general here. This will be left for future work.

Another important difference with Definition 3 is that the relative reading introduced there^12^ now becomes the base case for generic sentences. As noted above, in this respect we agree with the closely related proposal of Tessler and Goodman (2019)^13^. One might wonder what happened to the absolute reading that Definition 3 talked about? Does it disappear in the new approach? Not at all. We want to propose that the absolute reading now re-emerges as a special case of the interpretation rule given above. In case there are no salient alternatives to the group *G*, the factor in the equation that is due to these alternatives disappears and the assertability of generic sentences is entirely measured in terms of the conditional probability of *f* given *G*^14^,^15^.

As noted above, our proposal in this section is a special case of the causal analysis proposed in section 2.3. However, for the rest of the paper we will work with the somewhat simplified approach stated in Definition 5. This approach can account for the same examples that the proposal in section 2.2 can deal with. But we also get something extra. Taking a relative reading as the underlying and general meaning of generic sentences allows us, for instance, to account for the fact that the generic (9) seems false, or at least inappropriate in most situations. There is hardly any set of alternatives that would explain why there is anything special about Germans as far as right-handedness is concerned. On the other hand, talking about Germans seems to evoke very naturally comparison to other nationalities. So, it is hard to imagine a context in which such alternatives wouldn't be considered at all. But if such alternatives are salient, then the proposal above would predict the generic (9) to be not assertable.

(9)     ?Germans are right-handed.

The proposal also provides a way to understand the constraint ⋃*Alt*(*f*)⊈*G* we discussed to account for the oddness of examples like (10-a) and (10-b).

(10)     a.     ?Books are paperbacks.

      b.     ?Mammals are placental mammals.

According to this constraint these generics are odd, because the relevant feature (being a paperback) only applies to the targeted group (books). Assuming that generics are about distinguishing the group with the feature, together with well-established pragmatic constraints allows us now to make sense of this constraint. The pragmatic assumption we need is the Gricean rule that the sentence uttered needs to be informative. Notice that in the cases discussed here the fact that all objects with property *f* are part of group *G* is *a priory* knowledge: it is part of the meaning of these words. In other words, without observation you already know that all *f* are *G*. Therefore, the claim made by the generic according to Definition 5 that *f* is distinctive for *G* is not informative and, thus, out for pragmatic reasons.

## 4. Empirical Results on the Role of *G*-Alternatives

In the previous sections we have argued in favor of the claim that alternatives are relevant for the interpretation of a generic sentence of the form “*G*s are *f*” for several reasons: (i) alternatives to *f* are relevant to restrict the domain of the probability function; (ii) alternatives to the subject term *G* are relevant in case the generic has a relative, or contrastive reading, and (iii) alternative causal background factors influence our assessment of the extent to which (being a) *G* is causally relevant to *f*. Moreover, we have argued that alternatives to the subject term *G* are important in any case to learn the (inductive) generalization. We have motivated the importance of these sets of alternatives by looking at core examples in the literature. For the second set of alternatives we also provided independent evidence coming from the field of psychology of learning. In this section we will present the results of three empirical studies on the relevance of *G*-alternatives for the interpretation of generics. Ultimately, this should be done for the other sets of alternatives as well, but this will have to wait for future work.

### 4.1. The Hypotheses That We Will Test

The central goal of this part of our research was to empirically test whether alternatives to the subject term *G* do indeed affect the assertability of a generic sentence. Specifically, we hypothesize that the probability with which the alternatives carry the relevant feature *f* affects the assertability of the generic. This conforms with the account for generic sentences that we ended up with in section 3. According to this approach a generic *Gs are f* is the more assertable, the more distinctive the feature *f* is for the group *G*. The probability of *f* given *G* should be high *relative to* the probability of *f* given the salient alternatives to *G*^16^. **Hypothesis** 1. The assertability of a generic sentence “*Gs are f”* depends on the conditional probability of the feature *f* given salient alternatives *G*′ of *G*.

To test this hypothesis, we manipulate *P*(*f*|*G*′) and see whether we can observe an effect on the assertability of the generic. Depending on whether or not this hypothesis is supported by the data, we can then test different approaches to the meaning of generic sentences that explain the result. For instance, if the observed assertability is in line with Hypothesis 1, then we can evaluate the particular rule that we formulated in Definition 5 for the assertability of generic sentences. In other words, we can test whether contingency is a good predictor for the assertability of generic sentences.

**Hypothesis** 2. *The assertability of a generic sentence “*Gs are f”* is given by the formula*

$$Assertability\;of{\;}^{\prime }Gs\;are\;f^{\prime }=P\left(f|G\right)-P\left(f|⋃Alt\left(G\right)\right).$$

In the following, we will present the results of two experiments testing the hypotheses formulated above. We were looking for a setup that allowed us to probe the intuitions of people concerning generics about a group of objects for which they do not have any prior knowledge. This will allow us to ensure that participants do not have prior beliefs about features typical for the objects they will see. A second objective was to control the *G*-alternatives that the interpreters were considering. This is the factor that we will manipulate in order to see whether it influences the assertability of the generic sentence^17^.

**Figure 1:**
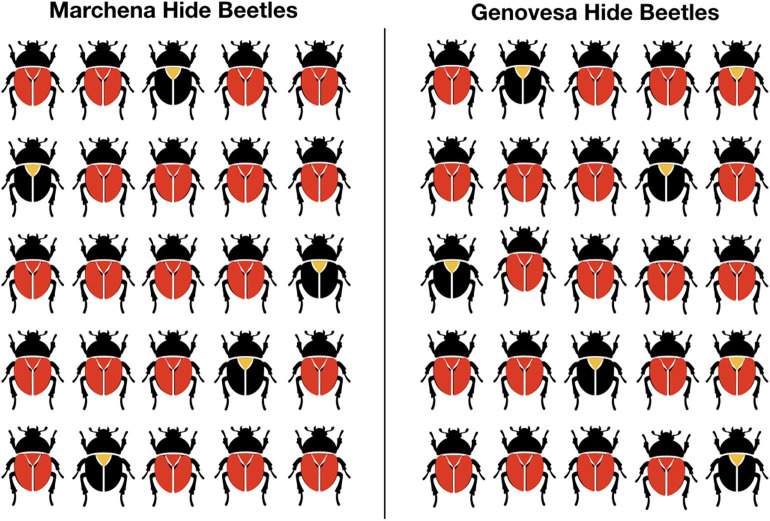
Sample picture in the non-contrastive condition with beetles.

We presented participants with a picture-sentence verification task similar to that used in Bordalo et al. (2016). The participants saw pictures with samples of fictive insect species from two Galapagos islands, Genovesa, and Marchena (see Figure 1)^18^. Their task was to assess whether animals from one of the islands, Genovesa, could be described with a given sentence. All sentences were generics stating that the species from Genovesa—our target group *G*—has a particular feature having to do with their coloring—our target feature *f*. We controlled the conditional probabilities *P*(*f*|*G*) that the participants of the studies assigned by manipulating how many of the animals *G* in the sample form Genovesa showed the particular coloring pattern *f*. The second sample from Marchena served as contextually salient alternative. By manipulating the frequency of insects with the relevant feature in this group we controlled *P*(*f*|⋃*Alt*(*G*)), from now abbreviated by *P*(*f*|*Alt*(*G*)).

**Figure 2:**
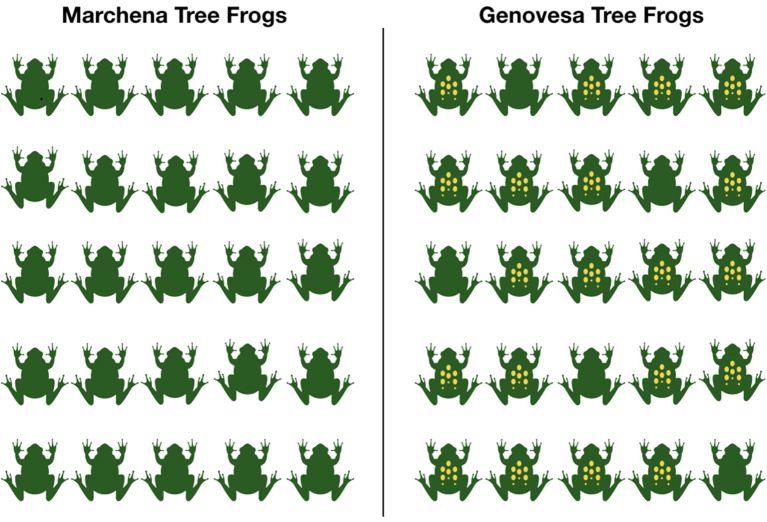
Sample picture in the contrastive condition with frogs.

We presented pictures in two conditions. In the non-contrastive condition an equal number of insects (80%) in both samples had the relevant feature *f* (see Figure 1). Thus, in this case *P*(*f*|*G*) = *P*(*f*|*Alt*(*G*)). In the contrastive condition, none of the insects in the sample from Marchena (the salient alternative) had the feature, while 80% of the insects from Geneva (the target *G*) had the feature *f* (see Figure 2). In other words, in this condition *P*(*f*|*G*) = 0.8 and *P*(*f*|*Alt*(*G*)) = 0. Based on Hypothesis 1, we expect that the strong difference of *P*(*f*|*Alt*(*G*)) between both conditions should have a significant effect on the assertability of the generic sentences. Hypothesis 2 predicts that the judgments of assertability people give for the generics should correspond to the contingency or the relative difference of feature *f* given group *G*.

### 4.2. Study 1

**Figure 3:**
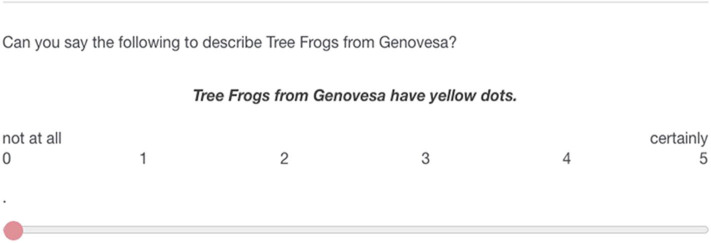
Example question from the study.

In the first study we used a within-subjects design. All participants gave an assertability score to one sentence in the contrastive condition, one in the non-contrastive condition and one filler sentence. Each question was presented with a different animal species (spiders, frogs, or bugs). Below the two samples, a generic sentence was given that always described the species from Genovesa. The participants were asked to judge on a scale from 0 to 5 whether the generic sentence was assertable given the provided data (e.g., “*Can you say the following to describe Tree Frogs from Genovesa?”*, see also Figure 3). They gave a response by dragging a slider as depicted in Figure 3. They could adjust their response with a accuracy of two decimals, so they experienced the scale as continuous.

Based on Hypothesis 1, we expected a significant difference in the judgments of assertability for both conditions. Hypothesis 2 claims that the judgments of assertability people give for the generics should correspond to the contingency of feature *f* given group *G*. In terms of proportions this measure predicts that the assertability of a generic should increase if feature *f* becomes more distinctive for the group *G*. Applied to the two conditions distinguished here we would expect that the generic is significantly more assertable in case of the contrastive condition than in the non-contrastive condition. The measure of contingency also makes precise numerical predictions for the assertability of generics. However, these predictions need to be translated into the scale presented to the participants in the study, because the range of the contingency function does not match the scale presented to the participants of the study: the contingency function ranges between −1 and 1, whereas the scale the participants saw let them grade the assertability of the sentences between 0 and 5. We used a linear transformation to map their responses directly onto the range [−1, 1] of the contingency function. Thus, 0 on the scale corresponds to a contingency of −1, 2.5 to a contingency of 0, and 5 to a contingency of 1. If we apply this linear transformation to the conditions that the participants of our study saw, Hypothesis 2 predicts that in the non-contrastive condition the contingency of the generic is 0, thus the participants should move the slide to around 2.5 on the given scale. In the contrastive case the contingency is *P*(*f*|*G*) − *P*(*f*|*Alt*(*G*)) = 0.8 − 0 = 0.8. This corresponds to the value 4.5 on the scale the participants saw. Given that there will be variation in how participants interpret the scale, we did not expect exactly the values predicted by the measure of contingency. However, the general proportional prediction described above should be visible in the data.

#### 4.2.1. Method

##### 4.2.1.1. Materials and procedure

We used pictures of three different animal species (Tree Frogs, Hide Beetles, Jumping Spiders). For each species we designed a picture in the contrastive and in the non-contrastive condition. All the pictures contained two samples, one with 25 animals of the species from Marchena, one with 25 animals from the species from Genovesa. For each species we had one corresponding generic sentence: “Hide Beetles from Genovesa have red wings,” “Tree Frogs from Genovesa have yellow dots,” “Jumping Spiders from Genovesa have green backs.”

The participants saw each animal species once, one in the contrastive condition, one in the non-contrastive condition and a third species as a filler. This resulted in three experimental trials per participant. In the filler condition, participants saw a generic that claimed the group to have a feature that none of the animals had (for instance, it could be the picture on Figure 1 with the generic “Hide Beetles from Genovesa have green wings”) and, therefore, this sentence was clearly not assertable. The filler condition was used to control whether participants completed the study in good faith: we excluded participants who gave a score above 1.5 in the filler condition as they likely did not pay attention in the other conditions either. The order in which the contrastive and the non-contrastive condition were shown was randomized. The filler always occurred last.

The study was implemented in Qualtrics. Participants started by reading the informed consent text and agreeing to taking part. They then read the instructions. Average time spent on the task was 143 s.

##### 4.2.1.2. Participants

Participants were recruited via Prolific.ac, an online platform aimed at connecting researchers and participants willing to fill in surveys and questionnaires in exchange for compensation for their time (Palan and Schitter, 2018). We recruited native English speakers (British and American English) who reported no vision impairments^19^. Eighty-two participants completed the task. Three participants were excluded: two because they did not give a response in one of the experimental items, one because they gave a score of 1.5 or above on the filler item. Thus, 79 responses were included in the analyses reported below.

Due to a mistake in the set up of the experiment, the participants were not forced to answer the filler questions. We therefore ended up with 27 participants who gave no response to the filler conditions. However, the slider was always at 0 by default, so these participants most likely simply agreed with the score 0 and therefore pressed “respond” without moving the slider. For this reason, we still included these participants in the analyses^20^.

#### 4.2.2. Results

The mean score given by the included participants in the filler condition was 0.04 (SD 0.16); the mean score in the contrastive condition was 3.51 (SD 1.06); and, finally, the mean score in the non-contrastive condition was 2.88 (SD 1.50). We performed a Bayesian paired samples *t*-test to test for the strength of evidence in favor of the null hypothesis (no difference between conditions) as opposed to the hypothesis that the score given by participants should be higher for contrastive than for non-contrastive condition using JASP software (JASP Team, 2018) with default priors. This analysis resulted in *BF*_10_ = 104, meaning that the data was 104 times more likely under our hypothesis than under the null hypothesis. Thus, the first study does lend support to Hypothesis 1 claiming that alternatives to *G* do affect the assertability of a generic sentence and the general prediction of Hypothesis 2 about the tendency of this dependency: comparing situations in which a feature is distinctive vs. ones where it is not distinctive for a group, the generic has a higher assertability in the situation in which the feature is distinctive.

In order to approximately evaluate compatibility of the observed scores with the predicted scores based on the Hypothesis 2, we investigated the 95% confidence interval (CI) around the mean in each condition, assuming a normal distribution. Note that the correct interpretation of 95% CI is that if we conducted our study multiple times with different participants and calculated a corresponding 95% CI for each group of participants, we would expect 95% of these confidence intervals to contain the true mean of the whole population. Thus, we expect that in 5% of the cases the confidence interval will not contain the true mean of the whole population. So it is possible that in our particular sample the CI does not contain this true mean. Note also that the assumption of normal distribution here is lenient. Given these considerations, the confidence intervals can give us only a rough idea of where the true value of the corresponding score in the population would lie.

We expected a mean score 4.5 in the contrastive condition, but observed 3.51 with 95% CI [3.27, 3.74] which does not include the expected score. For the non-contrastive condition, we expected a mean score 2.5, but observed 2.88 with 95% CI [2.54, 3.21] which again does not include the expected score, but does come close. Overall, while the scores come close to the expected ones, we cannot conclusively say that the observed values support our second hypothesis (but see the issues raised below in the *Interim Discussion* regarding the potential caveats of our approach).

**Figure 4:**
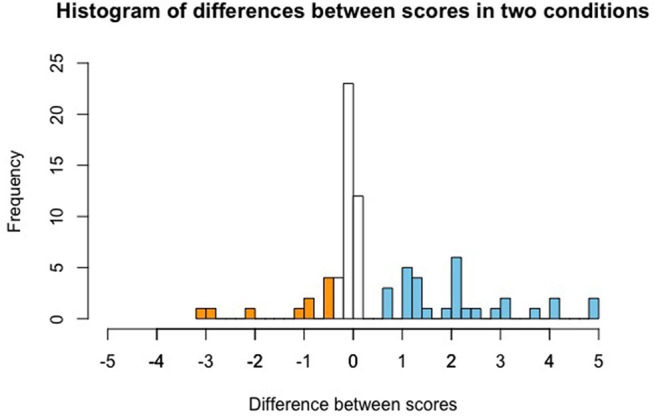
Histogram of differences in scores between conditions: contrastive condition minus non-contrastive condition. Differences below −0.5 are marked in orange color, differences above 0.5 are marked in blue color. Orange bars thus indicate participants who gave a higher score in the non-contrastive condition, non-colored bars indicate participants who gave a similar score in both conditions, and blue bars indicate participants who gave a higher score in the contrastive condition.

Figure 4 depicts the difference between given scores in the contrastive and non-contrastive conditions for each participant (specifically, displayed is score in contrastive condition minus score in non-contrastive condition). We can see that not all participants uniformly gave higher scores to the contrastive as compared to the non-contrastive condition. In fact, there was a sizable proportion of participants who gave approximately the same score in the two conditions, and even a small group that gave the non-contrastive condition a higher score than the contrastive condition. Thus, we seem to be observing different behavior patterns by different participants. We will come back to this in section 4.4.

### 4.3. Study 2

The results of Study 1 supported the hypothesis that the score given by participants to assertability of a generic sentence will differ for the case with an alternative present and the case with no alternative present. The generic “*Gs are f”* becomes in general more assertable in case the discussed feature *f* is distinctive for the group *G*. The results also partially support Hypothesis 2: in the non-contrastive condition the generic was judged to be in between assertable and non-assertable. In the contrastive condition the generic was on average rated to be assertable, though not to the degree predicted by the contingency measure. In order to replicate the original finding, we administered the same task in a between-participant set-up: each participant saw only one of the two conditions (contrastive or non-contrastive) plus a filler item.

#### 4.3.1. Method

##### 4.3.1.1. Materials and procedure

The materials used in this study were the same as in Study 1 except this time the participants saw only either contrastive or non-contrastive condition and a filler trial (2 trials in total). Average time spent on the task was 128 s.

##### 4.3.1.2. Participants

Participants were recruited via Prolific.ac with the same eligibility criteria. One hundred eighty-two participants completed the task. Three participants were excluded from the analysis because of a missing response to one of the items. Further seven participants were excluded because of giving a score above 1.5 in the filler question. That left 172 participants for further analyses.

#### 4.3.2. Results

The mean score given by the included participants in the filler condition was 0.07 (SD 0.23), in the contrastive condition 3.49 (SD 1.29; 95% CI [3.29, 3.68]), and in the non-contrastive condition 3.06 (SD 1.37; 95% CI [2.85, 3.26]). We performed a one-sided Bayesian independent samples *t*-test to test for the strength of evidence in favor of the null hypothesis (no difference between conditions) as opposed to the hypothesis that the score in the contrastive condition is higher than the score in the non-contrastive condition using JASP software with default priors. We obtained *BF*_10_ = 2.5, meaning that the data was 2.5 times more likely under the alternative hypothesis than under the null hypothesis. While this is not particularly strong evidence in favor of the alternative hypothesis, the data does show the same pattern as observed in Study 1. The diminished difference between conditions is likely due to that in Study 1, having two cases to compare, the participants noticed that the second set of objects changed (i.e., animals from Marchena), and this in turn strengthened the perceived contrast.

### 4.4. Interim Discussion

The results of both studies were in line with our Hypothesis 1: the probability of the feature *f* given a contextual salient alternative did affect the assertability of a generic sentence “*Gs are f”*. We also saw the direction of the dependence predicted by our theory supported: if *P*(*f*|*G*) is substantially larger than *P*(*f*|*Alt*(*G*)) then the assertability of the generic sentence is higher than in case there is no difference between both probabilities. We did not see the exact assertability scores that the theory predicts (Hypothesis 2). In the non-contrastive condition, the theory predicts an assertability of 2.5, while in Study 1 the average assertability in this condition was 2.88 and in the Study 2 3.06 with 95% confidence intervals around mean not including the expected value in either case. In the contrastive condition, we predicted an assertability of 4.5 and observed an average of 3.51 in Study 1 and 3, 49 in Study 2, again with the 95% confidence intervals around the mean not including the expected value.

Contrary to our expectation, the participants were not uniform in the scores they were giving—we observed large differences between participants' behavior, so in fact it does not make much sense to look at the overall means as we set out when we started this project^21^. However, this observation does not necessarily contradict the theory tested here. The predictions made by contingency as measure of the assertability of generic sentences depends on which alternatives to *G* the interpreter considers. We assumed that the setup of the study would lead the participants to consider the sample from Marchena as alternative to the sample from Genovesa that the generic talked about. The theory predictions outlined above are only valid if the participants took the alternative into account. However, we cannot be sure that the participants really did take the sample from Marchena to be a relevant *G* alternative. If they did not take any alternatives to the target group into account, the theory predicts the assertability of the generic sentence to be equal to the conditional probability *P*(*f*|*G*). Consequently, the assertabiility value assigned by the participants would be 4.

To explore this possible interpretation of the data, we separated the participants of Study 1^22^ into three groups: those that assigned the same assertability rating to the generics in both conditions (difference between scores in the two conditions <0.5^23^), those that judged the generic in the contrastive condition to be at least 0.5 points more assertable and those who considered the generic at least 0.5 points less assertable. 51% (*N* = 40) of the participants in the first study did not give a substantially different score in the two conditions, while 38% (*N* = 30) considered the generic in the contrastive condition more assertable than in the non-contrastive condition and 11% (*N* = 9) of the participants took the generic to be less assertable. We then looked at the scores given by participants in the first two groups^24^. If Hypothesis 2 is correct but only participants in the group that gave a higher score to the contrastive condition took the sample from Marchena as an alternative to the sample from Genovesa, these participants should have given the scores predicted by Hypothesis 2 whereas the participants in the group that did not take into account the sample from Marchena should have given score 4 in both conditions (as discussed above). This was not the case. In the group of participants that gave a higher score in the contrastive condition than to the non-contrastive position, the average assertability in the contrastive condition was 3.86 (SD 0.79; 95% CI [3.57, 4.14]) whereas the average assertability in the non-constrastive condition was 1.72 (SD 1.22; 95% CI [1.28, 2.15]). Thus, even in this subgroup of participants, the scores come close to the ones predicted by the theory, but we do not observe the exact values predicted by Hypothesis 2. The group that did not see a difference gave a mean score 3.35 (SD 1.18; 95% CI[2.98, 3.71]) in the contrastive and a mean score 3.4 (SD 1.22; 95% CI [3.02, 3.77]) in the non-contrastive condition.

There are a couple of remarks we want to add about the discrepancies between the assertability values predicted by the theory and the data obtained in the study. First of all, it is difficult to say how exactly the participants interpreted the scale that we asked them to use to indicate the assertability of the generic sentences they saw. We tried to avoid the ambiguity by labeling the extremes of the scale verbally as “not at all” and “certainly,” but cannot be sure what the participants did in case they were not sure about assertability of the sentence (when it is neither assertable nor non-assertable).

Depending on how the participants interpreted the scale, there might be also an issue with the way we interpreted the numerical values that our theory predicts (Definition 5). The range of the contingency function is the interval [−1, 1]. We took this to mean that −1 corresponds to a completely unassertable sentence, 1 to a sentence that is completely assertable and 0 describing the turning point from not assertable to assertable. This is how we translated the values of the contingency function to the scale that we presented to the participants of both studies. To some extend this is also supported by the data. The obviously wrong filler items got average assertability judgments that were very close to 0. However, there is no guarantee that even if the assertability of generic sentences can be described in terms of contingency, as we proposed, the values are interpreted in the linear manner that we assumed. Maybe a 0 for contingency already means that we wouldn't accept the sentence. To avoid such issues, we could show the participants a scale with numerical values from −1 to 1 instead 0 to 5 as we did here and see whether this affects their assertability judgments for the same set of test data. This will need to be taken up in the follow-up research.

To sum up, in general the results support the theory proposed here, though we did not see the exact scores that we expected. As discussed above, this could be because we did not transform the values from the theory to the scale seen by participants correctly. For this, more research in the future is necessary. What we can assess is in how far the theory explains the general tendencies in the data that we gathered, and in this respect the results are encouraging.

### 4.5. Study 3

The main goal of this final study was to test a different aspect of the theory developed in section 3.2. We repeat here for reasons of convenience Hypthesis 2, which contains the heart of the proposal.

**Hypothesis 2**. *The assertability of a generic sentence “Gs are f” is given by the formula*

$$Assertability\;of{\;}^{\prime }Gs\;are\;f^{\prime }=P\left(f|G\right)-P\left(f|Alt\left(G\right)\right).$$

So far, we have focussed on testing whether we can observe the predicted effects of manipulating the second argument of the measure of assertability. We saw that indeed *P*(*f*|*Alt*(*G*)) does affect the assertability of generic sentences and also that the kind of influence predicted (assertability goes up if *P*(*f*|*Alt*(*G*)) goes down) can be observed. In this study, we focused on the first part of the measure: *P*(*f*|*G*). Manipulation of this factor should, according to our theory, also have an effect on the assertability of a generic. Roughly put, increasing this variable should have a positive effect on the assertability ratings.

As a side question, we also wanted to test with this study whether another new aspect of our proposal can be supported by the data. As discussed in section 3.2, the approach introduced in Definition 5 also differs from the one described in Definition 3 in measuring the assertability of generics in degrees instead of proposing cut-off points that define the limit between being or not being assertable. For instance, if alternatives do not play a role, then Hypothesis 2 predicts a steady linear increase in the assertability of the generic with growing *P*(*f*|*G*). In some sense, the data of the first two studies already speak against a clear cut-off point of 0.5, given that even though *P*(*f*|*G*) was 0, 8 the assertability ratings were not close to ceiling^25^. Given that in this final study we consider different conditional probabilities *P*(*f*|*G*), the results should provide us with a clearer picture of whether the cut-off approach or the gradual change approach defended here come closer to reality.

In this last study, we used the same set-up as in the first two studies. The participants judged the assertability of generic sentences with respect to the two conditions, the non-contrastive condition in which *P*(*f*|*G*) = *P*(*f*|*Alt*(*G*)) and the contrastive condition in which *P*(*f*|*Alt*(*G*)) = 0. The only difference is that now we varied *P*(*f*|*G*) between participants.

As Study 3 was a follow-up to the first two studies, this time we assumed from the start that there will be two groups of participants. Participants that do not take alternatives into account when evaluating the generic sentence (we will refer to this group as *noCon*) are predicted to use the conditional probability of the feature *f* given the group *G* as measure of the assertability of the generic sentence. In this case, our theory predicts that in both conditions the assertability of the generic should increase linearly with a growing conditional probability *P*(*f*|*G*). For participants that *do* take the presented alternative into account (group *Con*) the assertability score should depend on *P*(*f*|*G*) and *P*(*f*|*Alt*(*G*)). In the contrastive condition, *P*(*f*|*Alt*(*G*)) is 0 while *P*(*f*|*G*) is not, so again the assertability of the generic sentence should grow linearly with the increase in *P*(*f*|*G*). Furthermore, we predict that the assertability ratings for this condition should overall be slightly higher (approximately 0.5 points) for the *Con* group than for the *noCon* group^26^. In the non-contrastive condition, both *P*(*f*|*G*) and *P*(*f*|*Alt*(*G*)) are identical so the contingency of the sentence is 0. In this case, for the *Con* group there should be no effect of proportion on the assertability of the generic sentence—the assertability score should be the same independent of *P*(*f*|*G*).

#### 4.5.1. Method

##### 4.5.1.1. Materials

This study had the same design as Study 1, but now we collected data for different proportions with which the animals possessed the relevant color feature. We used four proportions: 54, 68, 80, and 92%^27^. Furthermore, we also varied the distribution of the feature among the 25 animals that were shown to the participants: for each condition we used 3 pictures with different, randomly selected distributions of the feature over the presented animals.

Each participant had to make three judgments: she saw one picture in the contrast condition, one picture with the no contrast condition and one filler, all using the same frequency for the distribution of the feature. Each animal species was shown once. The order of the contrast/no contrast question was randomized, the filler was always shown as the third and last question^28^.

##### 4.5.1.2. Participants

Participants were again recruited via Prolific.ac with the same criteria. Four hundred and one participants completed the task. Twenty participants were excluded because they gave inadequate responses to the filler items (score above 1.5). Six further participants were excluded because they gave all three conditions a score 0. Three hundred and seventy-five participants were thus included in the analyses reported below: 97 for frequency 54%, 89 for frequency 68%, 94 for frequency 80%, and 95 for frequency 92%.

#### 4.5.2. Results

**Figure 5:**
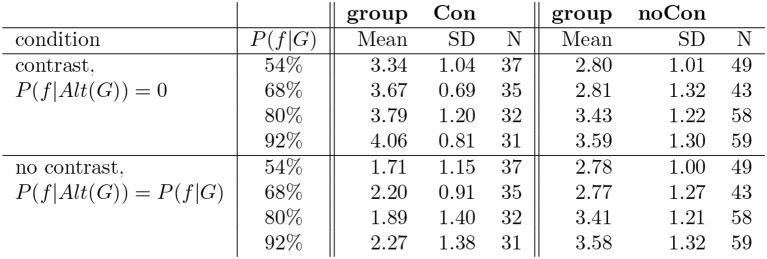
Results of study 3.

As stated above, in this study we distinguish two groups of participants: group *Con* contains participants that found the generic more assertable in the contrastive condition than in the non-contrastive condition; participants in group *noCon* did not give a different score in the two conditions. We split the participants into these two groups using the same criteria as we used in Study 1. There were 135 participants (36%) who gave a higher score in the contrastive condition (*group Con*). When collapsing across different proportions, this group gave a mean score 3.69 (SD 0.97) in the contrastive condition and a mean score 2.0 (SD 1.21) in the non-contrastive condition. There were 209 participants (55%) who gave the same score in the two conditions (*group noCon*). This group gave a mean score 3.2 (SD 1.26) in the contrastive and a mean score 3.18 (SD 1.25) in the non-contrastive condition. Finally, there were 18 participants (9%) who gave a higher score in the non-contrastive condition. The table in Figure 5 shows the results for the different probabilities split up according to the two groups that we distinguish.

To test our predictions, we conducted a Bayesian ANOVA with condition (contrastive vs. non-contrastive) and proportion (as an ordinal variable) as independent variables for each group separately. To evaluate whether a certain variable has an effect on the given scores, we compared a model including this effect with a model excluding this effect. For the group that gave the same score to both conditions (*group noCon*), we predicted an effect of proportion—the scores should linearly increase with increasing proportions. In the ANOVA analysis, we observed modest evidence against the effect of condition (*BF*_*Inclusion*_ = 0.2, given by the definition of the group), strong evidence for the effect proportion (*BF*_*Inclusion*_ = 13), and strong evidence against the interaction of condition and proportion (*BF*_*Inclusion*_ = 0.02). Thus, we do observe an effect of proportion. However, while the participants did give a higher score with increasing proportions, this increase does not seem to be equally present for all proportion steps. A *post-hoc* test comparing each proportion to the other ones showed that scores given for proportion 54% were not different from scores given for proportion 68% (*BF*_10, *U*_ = 0.16), and scores given for proportion 80% were not different from scores given for proportion 92% (*BF*_10, *U*_ = 0.22); for the other proportion pairs, we had evidence for the difference in scores. Thus, participants here did not seem to care about the difference between the lowest two proportions and the highest two proportions, exhibiting rather behavior that would correspond to there being some sort of threshold between *P*(*f*|*G*) = 68% and *P*(*f*|*G*) = 80%.

**Figure 6:**
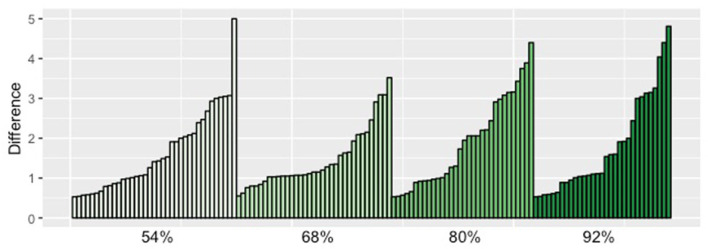
This plot depicts the difference between contrastive and non-contrastive condition (on the Y axis) for each of the 135 participants of the Con group (on the X axis). We grouped the participants by the proportion that they saw. We can see that it is not the case that there are mostly higher scores for higher proportions. NB: each participant saw only one proportion.

For the group that gave the contrastive condition a higher score than the no contrast condition (*group Con*), we predicted an interaction between condition and proportion: the scores given by participants should linearly increase with increasing proportions in the contrastive condition, but they should be the same across proportions in the no contrast condition. In the ANOVA analysis, we observed extreme evidence for the effect of condition (*BF*_*Inclusion*_ = ∞), inconclusive evidence for presence or absence of the effect of proportion (*BF*_*Inclusion*_ = 0.8) and modest evidence against the interaction of condition and proportion (*BF*_*Inclusion*_ = 0.2). Hence, based on our analysis, here the predictions were not borne out—the effects of condition and proportion did not clearly interact. When inspecting averages for each proportion in the two conditions, there *does* indeed seem to be a gradual increase of the scores in the contrast condition in this group, whereas in the no contrast condition there seems to only be a random fluctuation of the scores. But even if we focus only on the judgments for the contrastive condition, there is no evidence for an effect of proportion. It seems like the increase in scores was not consistently present for all participants (see Figure 6 for a depiction of the individual scores)^29^.

Because the proportion with *P*(*f*|*G*) = 0.8 is the same frequency of *f*'s given *G*'s that was used in Study 1, we can compare the results for participants that saw this proportion (*N* = 94) with the results obtained in Study 1. For this group, the mean score in contrastive condition was 3.50 (SD 1.25), whereas the mean score in non-contrastive condition was 2.88 (SD 1.47). When split into groups, there were 32 participants (34%) who gave the contrastive condition a higher score (difference more than 0.5) than the non-contrastive condition and 58 participants (61%) who gave them the same score (difference <0.5). Both the averages and the proportions of participants in each group are close to what we observed in Study 1. Hence, these findings are robust.

### 4.6. General Discussion

All three studies that we reported on support Hypothesis 1: the assertability of a generic sentence “*Gs are f”* depends on the conditional probability of the feature *f* given salient alternatives *G*′ of *G*. We also found evidence for the type of dependency predicted by our proposal made in section 3.2: if the feature *f* is much more frequent given *G* than given the alternative *G*′, then the assertability of the generic improves. Study 1 and study 2 did not support the exact assertability scores predicted by the theory, but as discussed in section 4.4, this might have to do with the particular methodology we used. In particular, our proposal for transformation of the scores in our task to those predicted by the theory might not be accurate.

With study 3, we wanted to investigate whether the predicted dependency on the absolute probability of *f* given *G* is also supported by empirical results. Based on the discussion in section 4.4, we now immediately distinguished two groups within the participants: group *Con* consisted of participants that judged the generic more assertable in the contrastive condition, while in group *noCon* were those participants that gave the same scores in the two conditions.

For the group *noCon*, the results of study 3 supported *a* dependence of assertability on proportion: the assertability increased with the probability *P*(*f*|*G*), independent of condition. But, as discussed above, we could not support the predicted linear increase in assertability that Hypothesis 2 predicts. Instead, there was some evidence for an assertability threshold between the second and third condition of proportion. This provides some evidence for threshold theories like the one of Cohen (1999), though the value of the threshold clearly seems to differ from the 50% threshold that Cohen proposes. Also the values below the threshold are not what one normally would expect. Even in the conditions with *P*(*f*|*G*) = 54% and *P*(*f*|*G*) = 68%, the generics still where not clearly rejected, but on average still marginally assertable. We need more empirical data, also for different conditions of proportions to be able to say whether we should prefer a threshold account and what form exactly it should take.

For group *Con* the data did not support an interaction between condition and proportion. Note that the mean assertability score given to the generic in the contrastive condition did steadily increase with growing conditional probability of the feature *f* given the group *G*, and in a rate that comes close to what is predicted by the theory. However, statistically the result was not significant. Here, either the theory is wrong or perhaps our experiment was not tapping into the interpretation/significance of alternatives clearly enough to reliably detect the difference. One reason for this could be that this effect (i.e., the increase in scores due to increasing *P*(*f*|*G*)) is rather small, so our sample size of approximately 30–35 participants in each group is not large enough to detect it. In this connection, notice also the surprising low assertability ratings of group *Con* for the non-contrastive condition. The theory predicts an assertability value of 0 in this case, independent of *P*(*f*|*G*), which should correspond to a score 2.5 on the scale the participants saw in our study (with our transformation). However, in study 1 and for all four proportions in study 3, the given assertability score was lower than that and varied quite a lot. We already discussed in section 4.4 that a possible explanation might lie in the way people interpreted the scale on which they gave their judgments.

Let us turn to the relevance of the data from the group *Con* for the cut-off point hypothesis built into theories like the one proposed in Definition 4 in contrast to the gradual increase in assertability that Hypothesis 2 predicts. As discussed above, for the group *noCon* there was some evidence for a cut-off point between *P*(*f*|*G*) = 0.68% and *P*(*f*|*G*) = 0.8%. In contrast, for the group *Con* we do not see the same “jump” in assertability ratings between proportions. Instead, as discussed above, at least in terms of just the means there appears to be a linear increase of assertability in the contrastive condition, as predicted by definition 5. From a theoretical point of view this observation is rather difficult to make sense of. Why should there be a cut-off point in case no alternatives are taken into account, while assertability increases linearly in case alternatives do matter? Of course, we could easily propose an ambiguity with two possible readings of generics; one with threshold, one without. But that seems to be an awfully arbitrary difference between two readings of the same sentences. Before we take such a theoretical step we need more evidence that this difference is real. To conclude, our results do not support a clear threshold account, as, for instance, defended in Cohen (1999). But also the linear increase of assertability with growing *P*(*f*|*G*) that Hypothesis 2 predicts is not completely supported by our data.

Finally, there is one more curious feature of the behavior of participants in study 3. Even though the few datapoints we recorded do not allow us to test for it, notice that the size of group *noCon* appears to increase with growing *P*(*f*|*G*). Using the terminology of our proposal, the higher the absolute probability of *f* given *G* the less relevant alternatives to *G* seem to be. There is some evidence from related domains, as studies of causal judgments, showing that actually *P*(*f*|*G*) counts more for the assertability of such judgements (Wasserman et al., 1993; Anderson and Sheu, 1995). Using a measure that takes this into account and, for instance, weighs *P*(*f*|*G*) more the larger this factor is, could explain the tentative observation just made. The higher *P*(*f*|*G*), the less the contrastive value *P*(*f*|*Alt*(*G*)) would count for assertability, and, hence, the smaller the difference between the contrastive and the non-contrastive condition. Consequently, more people would look like belonging to the group *noCon* instead of the group *Con*. Thus, if this tentative observation just made could be supported by a study suitable to test it, it might give us an important hint for how to improve the proposal made here.

Part of the problems we have with supporting the proposal tested here can be probably traced back to shortcomings with the particular experimental setting used here. We already mentioned in section 4.4 the issue with translating the experimental results into the scale of values predicted by the theory. One might be tempted to say that we should not aim at predicting exact assertability values. It is rather unusual for experimental psychology to formulate predictions in terms of specific scores as we did here, because it is assumed that there is too much uncertainty about what people are doing to have such precise predictions; traditionally, only presence or absence of differences between conditions is tested instead. However, we believe that formulating and testing more specific numerical predictions is a good way to reduce the gap between theories like the one about the meaning of generics presented here and experimental findings with human participants. But we also realize that methodologically this presents a number of challenges that we haven't solved completely yet.

**Figure 7:**
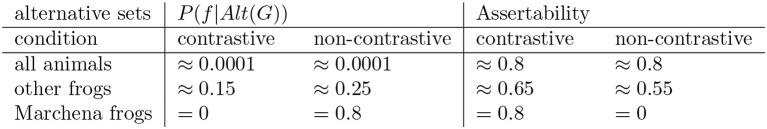
Assertability values for different alternative sets.

Another major issue with the setup we used is that it does not model sequential learning. A central idea of the theory proposed here is that assertability of a generic sentence is equated with the strength of association built based on the frequency with which the agent observed members of a group carrying a particular feature. However, we did not allow the learning of the association to observe these occurrences sequentially. Instead, we just gave the participants of the studies the information in one batch. But probably the limitation of the setup that had the most effect on the results obtained is the lack of control or insight in what the participants of the studies took the relevant alternative set to be. We assumed that the particularly setting used would entice the participants to take the corresponding species from Marchena, the species the other sample in each picture was from, as the only alternative to the target group: the species from Genovesa. But nothing in the experimental setting used made sure that this was indeed the case. The participants could have taken all kinds of alternative sets into account. Take, for instance, the example from the questionnaire used given in Figure 3. Maybe some participants of the study did indeed take the Tree Frogs from Marchena to be the only relevant alternative. But some might also have compared Tree frogs from Genovesa with what they know about frogs in general. Or they even considered all animals as possible alternatives. What they chose to be the relevant alternatives has, according to the approach tested here, a huge effect on how assertible they considered the given generic sentence about Tree frogs from Genovesa. In fact, this could account to a large extent for the huge variation we observed in the assertability judgements. Let us, as an example, just consider the alternative sets just mentioned and calculate the predicted assertability of the generic *Tree Frogs from Genovesa have yellow dots*. Thus, let *Alt*_1_(*G*) be the set of all animals, *Alt*_2_(*G*) be the set consisting only of frogs, and *Alt*_3_(*G*) be only Marchena Tree Frogs. First, we need to make assumptions about the prior probability of having yellow dots for each of the three potential sets of alternatives—again, this is something that different participants have different opinions about. Let's suppose for the moment that animals in general have only very rarely yellow dots, i.e., *P*(*f*|*Alt*_1_(*G*)) = 0.0001, frogs in general, however, tend to have yellow dots much more often, i.e., *P*(*f*|*Alt*(*G*_2_)) = 0.2. The participants won't have a prior for *Alt*_3_(*G*) because this is a novel species for them. Based on these priors, we can now calculate *P*(*f*|*Alt*(*G*)) after the participants saw the picture given in Figure 3. This information will hardly change anything for how probable one considers it that animals in general have yellow dots. But it will lower the probability for frogs having yellow dots, lets assume *P*(*f*|*Alt*_2_(*G*)) = 0.15. *P*(*f*|*Alt*_3_(*G*)) will be 0, based on the information in the picture. After seeing the non-contrastive counterpart of the picture in Figure 3 the respective probabilities *P*(*f*|*Alt*(*G*)) might be those given in Figure 7. These values would result in the assertability values given in the last two columns of the table. As the reader can see, there are huge differences between the various assertability values. For instance, a speaker who takes all animals to be relevant alternatives to the observed species would not see a detectable difference between both conditions, but would take the assertability in both cases to equal the conditional probability *P*(*f*|*G*). Taking a smaller set of alternatives results in some difference between both conditions and a generally lower assertability in both conditions. Taking actually only the species from the alternative island to be a relevant alternative results in the extremely different assertability values that we expected.

This shows first of all that the distinction between participants that do and participants that don't take alternatives into account, which lies at the bottom of the way we analyzed the data of the second study, is not the only way to explain the substantial group of participants that don't see a difference between both conditions. These might also be participants that just consider a very general set of alternatives. Second of all, we have here a way to explain the substantial variation in the data from the perspective of the proposal made. It also points, as said at the beginning of this discussion, to a major weakness of the experimental setup used here. In order to truly test the proposal at hand we need to either control, or probe what the participants of the experiments take to be the relevant alternatives. This will be a focal point of our future work.

## 5. Conclusions

The main goal of this paper was to explore and defend a statistical approach to the meaning of generic sentences. Such approaches are in discredit at the moment, because of the various shortcomings of the majority rule, which is the most popular statistical approach to generics on the market at present. However, we think that there is a vast variety of different statistical approaches to the meaning of generics that have a lot of promise. In this paper, we discussed in particular that by taking into account various notions of alternatives for the interpretation of generic sentences, many shortcomings of the majority rule can be overcome. In particular, we argued that alternatives are relevant to the meaning of generics in three different ways. We have seen that alternatives of the property *f* that the generic ascribes to group *G* matter, as well as the alternative causal background factors. Finally, we saw that also alternatives to the group *G* matter for the acceptability of the generic. This has led us to a first and preliminary formal description of the meaning of generic sentences, given in Definition 3.

We then zoomed in on the alternatives to the group *G* the generic is ascribing some property *f* to. We motivated the relevance of these alternatives for the meaning of generics by linking this meaning to associative learning. Building on theories of learning from psychology, we formulated a new and final version of our approach. According to this proposal, essential for the assertability of a generic sentence *Gs are f* is how distinctive the feature *f* is for the group *G*. We have motivated this approach on the one hand by showing that it can account for many problematic examples in the literature, and on the other hand by showing that such an approach can be motivated by considerations for the psychology of learning and results on the link between statistical information and causal dependence. In short, distinctiveness matters for the assertability of generic sentences, because this condition is essentially linked to how we learn about causal dependencies in the world. This proposal differed from the approach we formulated at the end of the first part of the paper in two important respects. First of all, it predicts the assertability of generics to come in degrees. More concretely, this means that our proposal does not assume strict cut-off points for the truth or assertability of generics. Secondly, the proposal assumes not two, but only one (context-dependent) reading for generic sentences. This reading is the relative reading of Definition 3. The reading can in certain circumstances—if the alternative set the interpreter assumes for *G* is empty—collaps to the absolute reading of Definition 3.

In the final section of the paper, we reported on three studies that tested our final proposal. In these studies participants were presented with a visual scene and asked to judge the assertability of a generic sentence *Gs are f* . We manipulated the presence of the alternatives and the frequency with which members of group *G* carried feature *f*. The results allowed us to confirm the relevance of *G*-alternatives for the meaning of generic sentences in the population in general. We also observed some evidence for the correlation between assertability of generic sentences and *P*(*f*|*G*). However, not all particular predictions made by the proposal in section 3.2 were borne out.

We also saw that the experiment setting explored here still has a number of shortcomings. Two should be the focus of future work along the lines explored here. First of all, we need to develop an experimental paradigm that allow us to test the link made here between the assertability of generic sentences and learning more directly. In particular, we need to model learning more naturally in the experimental setting. The second is to find a way to gain more insight or control on what the speaker of a generic sentence takes to be the relevant alternatives. As we have seen in the last part of the previous section, assuming that there was a lot of variety of what the participants of the studies took to be the relevant *G*-alternatives can account for huge variation in the assertability judgements observed. In future work we need to invest in experimental methods that allow us to probe or manipulate these alternatives sets.

Though the most pressing challenges for future work on the topic explored here are arguably methodological in nature—we need a solid empirical basis in order to direct further theoretical work—there are also a couple of interesting theoretical questions that we want to explore in future work. Just to mention one example, we picked contingency to measure associative learning. However, causal impact was not tested and there are also other measures of strength of association discussed in the literature. We should test those as well on the data-set gathered here and compare the predictions made with those of contingency.

## Data Availability Statement

The raw data and analysis script are available on https://osf.io/hyt8d/, doi: 10.17605/OSF.IO/HYT8D.

## Ethics Statement

Ethical review and approval was not required for the study on 2022 human participants in accordance with the local legislation and 2023 institutional requirements. The participants provided their informed consent to participate in this study.

## Author Contributions

All authors listed have made a substantial, direct and intellectual contribution to the work, and approved it for publication.

## Conflict of Interest

The authors declare that the research was conducted in the absence of any commercial or financial relationships that could be construed as a potential conflict of interest.

## Footnotes

^1^Although such an analysis seems natural, it is not the strategy that Cohen (1999) suggests to account for examples like (3-a) and (3-b). Instead, Cohen (1999) proposes that these type of sentences should be treated as relative readings, to be discussed in the next section.

^2^According to one of them, what counts is not whether, for (7-a) for instance, the majority of Dutchmen *actually* are good sailors, but whether they *can* or *would* be good sailors if they tried. Although such a strategy might look appealing, the strategy seems to over-generate enormously: why, then, is an example like “Children are dangerous” not true, just because these children *can* be dangerous? According to another strategy, one might say that these sentences are actually false. But why, then, do so many people take them to be true? A major worry here is to determine what the data are: if (7-a)–(7-b) are generally taken to be true, what is it that makes the claim “correct” that these sentences are *in fact* false? It cannot be that this is so because it is predicted by the theory, because the theory itself is based on intuitions of the language users.

^3^Forgetting for simplicity now about *Alt*(*f*).

^4^By taking other large mammals as alternatives.

^5^In contrast to Cohen (1999) we will in this section interpret a conditional probability like *P*(*f*|*G*) as ranging over open formulas. Thus, *P*(*f*|*G*) really measures the amount of *G*s that are also *f*. Cohen (1999) rightly observes that in this way the “unbounded” character of generics cannot be accounted for. We agree, but we will propose our own remedy to solve this problem.

^6^Leslie (2008) wonders how a proponent of a probabilistic account can explain why the generic “Bees reproduce” seems true, while “Bees are sterile” is false. The problem is that if “Bees are sterile” is (correctly) predicted to be false because it is not the case that the conditional probability *P*(*Sterile*|*Bee*) is high for all types of bees, the generic “Bees reproduce” is for that reason (wrongly) predicted to be false as well, because members of at least one type of bee (the workers) don't (tend to) reproduce. We think that “Bees reproduce” is nevertheless true, because in many cases plurals like “bees” and “ants” are seen as mass nouns and have a *collective* interpretation due to the fact that these are very small insects that we most of the time don't individuate (cf. van Rooij and Schulz, 2020a). On such a (semi-) collective interpretation of “Bees reproduce,” it doesn't have to be the case that all (minimal) subgroups of bees reproduce, it is only required that the whole group—or better, larger subsets of this group—does so. Notice that although in English, “bee” and “ant” are count nouns, their counterparts in languages such as Welsh (Stolz, 2001) and Dagaare (Grimm, 2009) are actually mass nouns. This suggests that it is at least natural to view bees and ants primarily as collections.

^7^To be sure, we don't think that all generics have such a causal interpretation, but we think that many of them have.

^8^This is the way Pearl (2000) estimates *P*(*C*|*do*(*A*)) when no explicit intervention, or experiment, is possible. *B* is thought of as the confounding variable that should be controlled.

^9^In van Rooij and Schulz (2019, 2020b), a slightly different notion is used, the notion of ‘probability of causal sufficiency'. One can show that under some natural conditions this comes down to $\frac{P\left(f|do\left(G\right)\right)-P\left(f|do\left(\neg G\right)\right)}{1-P\left(f|\neg G\right)}$ – which is basically the same as Cheng's (1997) notion of *G*s ‘causal power' to produce *f*. Although to determine the numeral value of causal power, the denominator is important, we will ignore this denominator in this paper.

^10^Take *c*_*i*_ to be *c*_1_. Then it could be that the actual cue was *c*_1_*c*_2_ and that *V*_*n*_(*o*|*c*_1_*c*_2_) = 1, although *V*_*n*_(*o*|*c*_1_) would be 0.

^11^We take it to be very natural, however, that people take the associations, to be the conditional likelihood. In fact, according to, e.g., Newel et al. (2007), we can explain many of the problematic probability judgements as found in, e.g., Tversky and Kahneman (1974) by the assumption that people confuse probabilities with associations as established via associative learning mechanisms like (RW). See van Rooij and Schulz (2020a) for a use of this idea for the analysis of generics.

^12^According to Cohen (1999) this is the less important reading of generics.

^13^Although they don't base or motivate their proposed analysis on learning-theoretic grounds.

^14^If one assumes that *P*(*f*|*X*) = 0, if *X* = ∅, this straightforwardly follows from Definition 5. For a more principled motivation, see Cheng's (1997) and van Rooij and Schulz (2019).

^15^Notice that this still doesn't mean that the assertability of generics does come down to the majority rule in this case. We keep the claim that assertability comes in grades (the grade is given by the conditional probability *P*(*f*|*G*)) and don't assume a cut-off point of 0.5.

^16^We want to emphasize that the feature that we are looking for here: the relevance of distinctiveness for the meaning of generic sentences, is in itself not distinctive for the particular approach we are defending here. A similar prediction is also made by approaches to generics that take them to be at the core assertions about kinds and thereby link them to how we represent kinds (Leslie, 2008). As, for instance, argued in Cimpian et al. (2010b) such an approach predicts that “…features that are privileged in our concepts may be more acceptable than generic predications of features that are not, all other things being equal.” (Cimpian et al., 2010b, p. 1,456). Thus, “the more striking, appalling, or otherwise gripping we find the property predicated in the generic, the more tolerant the generic is to exceptions” (Leslie, 2008, p. 15). Distinctiveness is taking to be one of the characteristics that makes a property more gripping. We come to the same predictions about the relevance of distinctiveness, but via a different route. In our case it is linked to the learnability of the expressed causal dependence.

^17^This is not the first time the hypothesis that distinctiveness matter is empirically tested, see in particular (Cimpian et al., 2010b). Also in this paper novel categories are used to test the impact of contrastiveness on generics. They, however, ask people to judge the truth of a generic sentence, while we focus on assertability. Furthermore, they present the prevalence of the relevant feature in the target group verbally, while we provide this information graphically. We think that this is a more natural setting given the connection we want to make to learnability (though also our paradigm has its limitations, as we will discuss later). Furthermore, our experimental paradigm makes a much more fine-grained and precise manipulation of distinctiveness possible.

^18^The names of the islands are real. The participants were also shown a map of the Galapagos islands with the location of the islands. We chose animals instead of, for instance, manipulating the clothing of people, because the coloring of animals would not be perceived as an accidental feature of the observed individuals.

^19^Since the material involved colors, the participants were required to have normal vision of colors.

^20^Excluding these participants did not make a difference to the results reported here.

^21^Note that we report the mean values and statistics with the whole group despite this since we committed to an analysis plan before we collected data.

^22^This was not possible for the second study since we used a between-participants setup in that case.

^23^This is an arbitrary threshold that we chose. We assumed that a difference of 0.5 could arise from the participants trying to drag the slider to the same point on the scale, whereas larger differences would necessarily arise from intentional positioning of the slider at different points of the slider.

^24^We will not discuss the participants in the third group which gave the non-contrastive condition a higher score than the contrastive condition further as we do not know why they behaved like that. They could have not understood the instructions or they could have changed their interpretation of the target sentence halfway through the experiment.

^25^Cohen could argue that this is because some or all of the participants applied the relative reading of generics. However, notice that even after we split participants into groups according to whether they saw a difference between the two conditions, those that did not see a difference still did not give a ceiling assertability score to the generic sentence. Furthermore, in the relative reading, Cohen would predict that still the generic should be completely assertable in the contrastive condition and completely unassertable in the non-contrastive condition, which is again not what we found.

^26^The reason for this is a difference in how *P*(*f*|*G*) counts for assertability for participants that take alternatives into account and those that don't. The assertability rating of a participant that doesn't take alternatives into account in the condition where 80% of the animals carries the relevant feature, for instance, should be *P*(*f*|*G*)*5 = 4, 0. But a participant that takes alternatives into account should give in the contrastive condition a rating of $\frac{P\left(f|G\right)+1}{2}*5=4,5$.

^27^All sample-pictures contained 25 animals of one species (see Figure 3). Thus, for example, in the contrastive condition a proportion of 54% means that 14 out of 25 animals in the sample from Genovesa have the property and none of the animals in the sample from Marchena. In the non-contrastive condition in both samples 14 out of 25 animals would have the property.

^28^As a consequence, the trials using 80% were a complete replication of the first study. We will come back to this in the discussion of the results.

^29^The reader might notice that in the 54% condition only one participant had a very large difference - 5, and there is no other participant in other proportions with such a large difference between the contrastive and the non-contrastive condition. One might think that maybe this participant is the reason why we do not observe an effect of proportion. But excluding this participant does not affect the results.
